# Influence of Basalt Fibers on the Crack Resistance of Asphalt Mixtures and Mechanism Analysis

**DOI:** 10.3390/ma15030744

**Published:** 2022-01-19

**Authors:** Bangwei Wu, Weijie Meng, Ji Xia, Peng Xiao

**Affiliations:** 1 College of Architectural Science and Engineering, Yangzhou University, Yangzhou 225127, China; wubw@yzu.edu.cn (B.W.); MX120190454@yzu.edu.cn (W.M.); MZ120200952@yzu.edu.cn (J.X.); 2 Research Center for Basalt Fiber Composite Construction Materials, Yangzhou University, Yangzhou 225127, China

**Keywords:** basalt fiber, asphalt mixture, crack resistance, environmental scanning electron microscope

## Abstract

The paper aims to investigate the influence of basalt fiber (BF) on the crack resistance of the asphalt mixture and conduct a mechanical analysis. First, two typical asphalt mixtures, namely AC-13 and SMA-13, were designed. The impact of BF on the mixture design results was analyzed. Then, several macroscopic tests, namely the four-point bending test, indirect tensile test, and semicircular bending test (SCB), were conducted to assess the effect of BF on the cracking resistance of asphalt mixtures. Finally, the influence of BF on the cracking resistance of asphalt mixtures was analyzed based on an environmental scanning electron microscope (ESEM) observation. The results show that: (1) BF increases the optimal asphalt content of AC13 and decreases the optimal asphalt content of SMA-13, which is caused by the different asphalt-absorption capacity of BF and lignin fiber (LF). (2) BF enhances both the fatigue crack resistance and temperature crack resistance of asphalt mixtures. The enhancement on the SMA-13 is more significant, indicating that the enhancement of BF on asphalt mixtures is related to the type of aggregate gradation. (3) BFs in the asphalt mixture lap each other to form a spatial network structure. Such structure can effectively improve the crack resistance of the mixture by dispersing the load stress and preventing the flow of asphalt mastic. The study results provide an effective method to design crack-resistant asphalt mixtures.

## 1. Introduction

Complex climates and increasing traffic loads have a severe negative impact on the cracking resistance of asphalt pavements [1]. Engineers, therefore, have tried to improve the performance of pavements with external admixtures [2,3]. Fibers and polymers are the two main admixtures for asphalt mixtures. Fibers have already been applied for asphalt mixtures for more than 50 years [4]. After the 1990s, with the use of stone mastic asphalt (SMA) in pavements, more fibers were adopted in asphalt mixtures [3], such as synthetic fibers [5], kenaf fiber [6], glass fiber [7], etc. Scholars studied the influence of fibers on the asphalt mixture performance, and concluded that fibers can effectively improve mixture performances [8,9]. In recent years, basalt fiber (BF), a green and high-performance material, has received increasing attention. BF is made of natural basalt stone. It is made by quickly drawing the molten basalt lava at 1400–1500 °C. No other chemical additives are used in the production process, and there is no wastewater, gas, or slag discharge [10,11]. Therefore, BF is an environment-friendly fiber. Many researchers have tried to use basalt fibers in asphalt mixtures. Morova investigated the usability of basalt fibers in hot mix asphalt mixtures. Based on the Marshall stability test results, he found that the best fiber ratio was 0.5% by the mass of asphalt mixtures [12]. Celauro used basalt fibers for urban asphalt pavement. He argued that basalt fibers proved to satisfy specific needs in road technique, and fibers were likely to slightly increase the micro-texture of asphalt mixtures [13]. Zhang used a three-dimensional fiber distribution model to investigate the effect of BF distribution on the flexural–tensile rheological performance of asphalt mortar. He argued that the flexural–tensile rheological value under the horizontal-oriented fiber is minimum [14]. Katharine et al. [15] investigated the microstructure of basalt fibers and the fibers’ asphalt mastic at different magnifications with scanning electron microscopy. The finding shows that the fibers are randomly distributed in the asphalt mixtures.

The crack resistance of asphalt mixtures has been considered a significant factor in the design of asphalt pavements. According to the report from the National Cooperative Highway Research Program (NCHRP) 9–57, cracks in asphalt mixtures were divided into four categories: temperature-related cracks, load-related cracks, fatigue cracks, and reflection cracks [16]. Many methods have been developed to determine the cracking resistance of asphalt mixtures. Zhou suggested an IDEAL cracking test for asphalt mixture design. He believed this method was sensitive to RAR content, asphalt content, aggregate gradation, and other variables [17]. Turipan used many indicators based on semi-circular bending (SCB) tests to evaluate factors affecting the cracking resistance of asphalt mixtures. He argued that the Flexibility Index (FI) was more suitable than peak load to judge the cracking property of asphalt mixes [18]. Yan compared SCB-IFIT, un-notched SCB-IFIT, and IDEAL-CT for measuring the cracking resistance of asphalt mixtures. He found that IDEAL-CT has a high correlation with SCB-IFIT but with a lower variation in results [19]. Poulikakos [20] compared and analyzed the four-point bending (FPB) and cantilever beam two-point bending fatigue test methods and found significant differences between the two results. He suggested four-point bending to evaluate the fatigue performance of asphalt mixtures. Hasan [21] compared the difference between four-point bending fatigue and semi-circular bending tests and found that four-point bending fatigue is better for distinguishing coarse and fine mixes. There are many more studies on the cracking performance testing of asphalt mixtures [22,23,24], and it is difficult to cover them all for the length of this article. In summary, it can be found that it is hard to evaluate different cracking using a single test method, because different cracking mechanisms cause different types of cracks. For example, Islam argued that the current fatigue model in the Mechanistic-Empirical (ME) Design Guide was incomplete due to the fact that the temperature-induced fatigue damage was not considered [25]. In his study of the cracking properties of fiber-reinforced polymer matrix composites, Budiman pointed out that the addition of fibers may alter the way the matrix cracks [26]. Thus, for the fiber-reinforced asphalt mixtures, multiple test methods are needed to fully evaluate their cracking properties.

The incorporation of fibers into asphalt mixtures has gained more and more attention. Many researchers argued that fibers help to improve the cracking resistance of asphalt mixtures. On the other hand, cracking resistance is rather critical when it comes to the design of asphalt mixtures [27]. As a new fiber, BF’s effect on the cracking resistance of asphalt mixtures has not been explored enough. The suitability of BF for use with different graded asphalt mixtures has also not yet been studied. Thus, to further evaluate the effect of BF on the cracking resistance of asphalt mixtures, this paper designed two typical asphalt mixtures and used three tests to determine the cracking performance of the asphalt mixtures. An environmental scanning electron microscopy (ESEM) was also used to analyze the enhancement mechanism of BF. The conclusions of this study help to improve the crack resistance of asphalt mixtures and understand the reinforcement mechanism of fiber asphalt mixtures.

## 2. Materials and Asphalt Mixture Design

### 2.1. Materials

#### 2.1.1. Asphalt

SBS-modified asphalt is widely used in China for asphalt pavements. The asphalt used in the research is SBS-modified asphalt, and the main technical properties of SBS asphalt are shown in Table 1. Its technical properties met the JTG F40-2004 Technical Specification for Construction of Highway Asphalt Pavements requirements.

#### 2.1.2. Fibers

**Figure 1:**
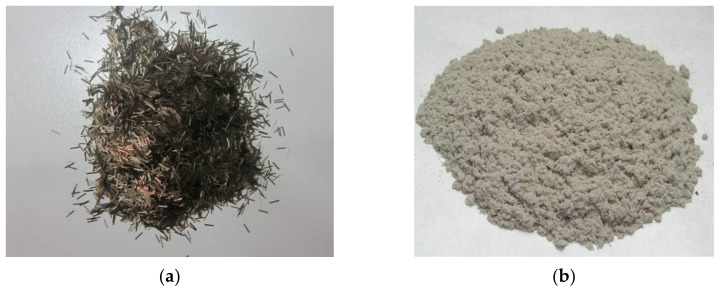
The appearance of the two fibers: (**a**) BF and (**b**) LF.

Basalt fiber (BF) and lignin fiber (LF), two types of fibers, were used in this experiment. The appearance of both fibers is as shown in Figure 1, and their basic properties are shown in Table 2. LF was only used for SMA-13. BF was used for both SMA-13 and AC-13.

#### 2.1.3. Mineral Aggregates

Two types of natural stone, limestone and basalt, were used for asphalt mixture design. The aggregates were within four size ranges, namely 10–15, 5–10, 3–5, and 0–3 mm, respectively. The basic properties of the aggregates are shown in Table 3.

### 2.2. Asphalt Mixture Composition Design

**Figure 2:**
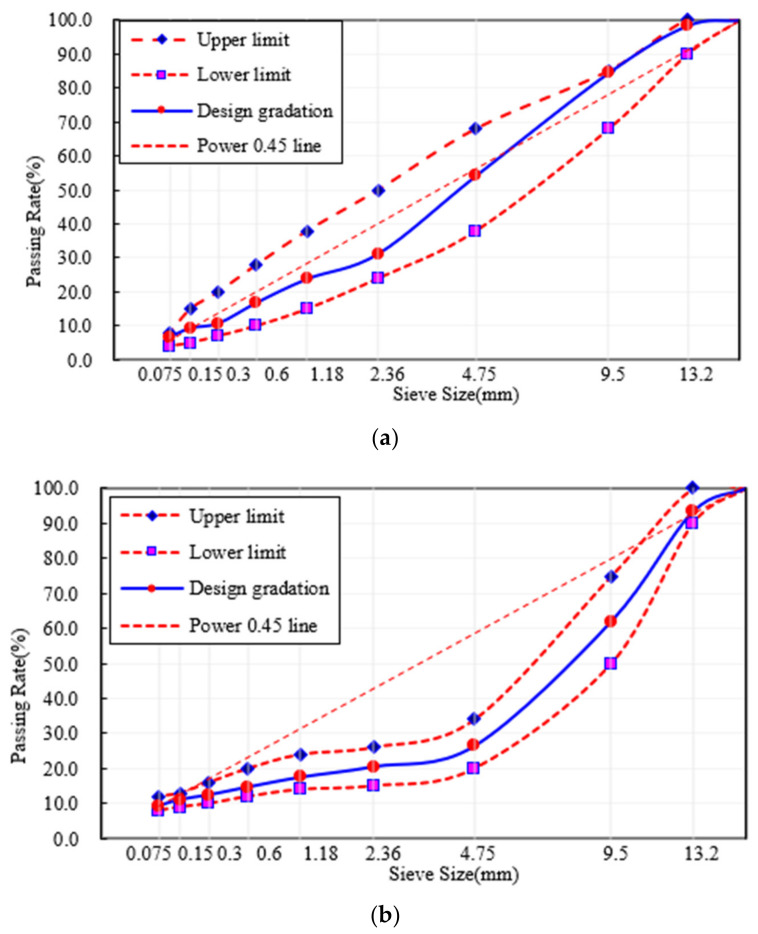
Aggregate gradation: (**a**) AC-13 and (**b**) SMA 13.

AC-13 and SMA-13 were designed with the Marshall method for this study. The two mixtures are the most widely used in China. The nominal maximum particle size of aggregates is 13.2 mm. The aggregate gradations of AC-13 and SMA-13 are shown in Figure 2.

Four asphalt mixtures, namely SBS AC-13, SBS+BF AC-13, SBS+LF SMA-13, and SBS+LF+BF SMA-13, were prepared. The BF constitutes 0.4% of the total asphalt mixture mass in AC-13 (BF 0.35% of the volume of AC-13). In SBS+LF+BF SMA-13, BF and LF account for 0.3% and 0.1% of the total asphalt mixture mass, respectively (BF 0.29% of the volume of SMA-13, LF 0.2% of the volume of SMA-13). In the control group of SBS+LF SMA-13, LF accounts for 0.3% of the whole asphalt mixture mass (LF 0.61% of the volume of SMA-13). Such fiber contents are based on the Chinese specification T/CHTS 10016–2019 Technical Guideline for Construction of Asphalt Pavement with Basalt Fiber.

For the fabrication of fiber asphalt mixtures, a “dry mix” process was used. The fibers were first blended with the aggregate for 90 s. Then, the asphalt was added to ensure the uniform dispersion of fibers in the asphalt mixture. The volume parameters of the four asphalt mixtures are shown in Table 4.

As shown in Table 4, BF increases the optimal asphalt content of AC-13 and decreases that of SMA-13. BF has a specific asphalt absorption capacity, so the addition of basalt fibers to AC-13 causes an increase in optimal asphalt content. However, the BF has a lower asphalt absorption ability than LF, and the amount of LF in SBS+LF SMA-13 is more than that in SBS+LF+BF SMA-13, causing the optimal asphalt content of the latter to be lower.

## 3. Test Methods

### 3.1. Four-Point Bending Fatigue Test

The four-point bending fatigue test was carried out in line with the AASHTO T 321 specification [28]. A formed asphalt mixture slab (size 4000 × 3000 × 80 mm) was cut into beams of size 380 × 63.5 × 50 mm. Four beams can be formed per asphalt mixture slab. This specimen was tested in the UTM (universal testing machines) software operating system with the test temperature of 15 ± 0.5 °C and the specimen preloaded at the target stress level for 50 cycles. The strain levels for this study were 450, 650, and 850 με. The test index represents the number of fatigue actions with accumulated dissipated energy.

### 3.2. Indirect Tensile Test

Indirect tensile tests were carried out according to AASHTO T322 [29]. The specimens were formed with the standard Marshall compaction method. The specimens were tested in a UTM-25 test machine at a temperature of 15 ± 0.5 °C. The test procedure will be terminated when the specimen reaches a vertical deformation value of 10 mm. Tensile strength and toughness index (*T_I_*) are used as test indicators. *T_I_* is calculated according to Equation (1), where *A_d_* is the area of the lower curve corresponding to *d/d_p_* after dimensionless processing, *A_p_* is the area of the lower curve corresponding to the peak after dimensionless processing, *d* is any deformation value greater than *d_p_*, and *d_p_* is the deformation value corresponding to the peak load:

(1) $$T_{I}=\frac{A_{d}-A_{p}}{d/d_{p}-1}$$

### 3.3. Semi-Circular Bending Test (SCB)

The SCB test was conducted in strict accordance with the AASHTO TP105 [30]. This test was conducted to determine the crack expansion performance of the asphalt mixture at a room temperature of 25 °C with a vertical displacement of 50 mm/min applied. It requires a pre-cut joint of a certain length at the bottom of the specimen in advance. The main evaluation indicators for this test are the fracture energy (*G_f_*) and the flexibility index (*FI*). *G_f_* is the fracture energy, and it is calculated according to Equation (2), where *W_f_* is the integral of the load-displacement curve, and *Area_lig_* is the ligament area and the thickness of the specimen (*t* = 50 mm). The *FI* was adopted to reflect the crack propagation rate, and it was calculated using Equation (3), where |m| is the absolute value of the slope at the inflection point after the peak of the loading value. The *FI* value is negatively correlated with the crack propagation rate. Four specimens were used to determine the *FI*.

(2) $$G_{f}=\frac{W_{f}}{Area_{lig}}\times 10^{6}$$

(3) $$FI=\frac{G_{f}}{\left|m\right|}\times 0.01$$

### 3.4. Environmental Scanning Electron Microscope (ESEM)

To study the distribution of basalt fibers in the asphalt mixture and the bond between the fibers and the asphalt mastic, the ESEM was used. The microstructural features of the damaged surface of samples that were taken from indirect tensile fractures were observed with ESEM to analyze the nature of the mechanical contribution of BF to the asphalt mixture.

**Figure 3:**
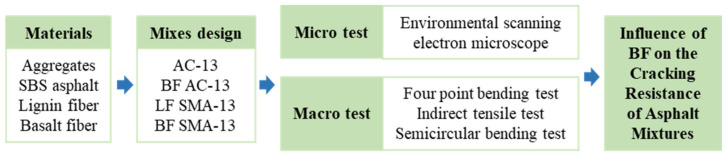
Flow chart of this study.

The main flow chart for this study is shown in Figure 3.

## 4. Results and Discussion

### 4.1. Four-Point Bending Fatigue Test Results

**Figure 4:**
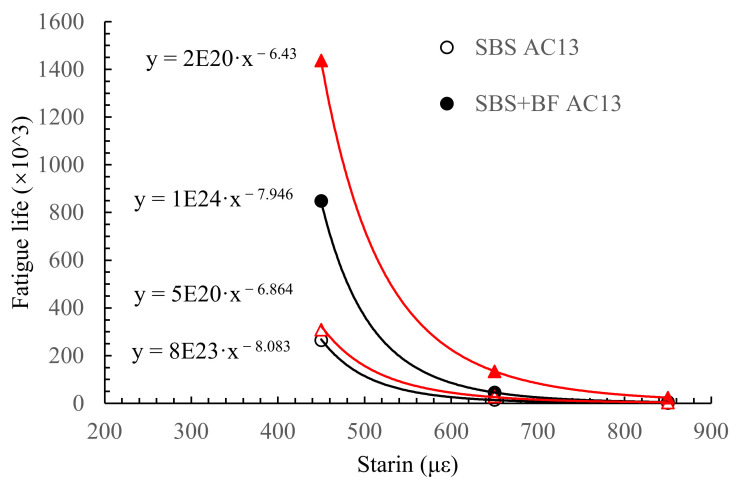
Fatigue life of the asphalt mixtures.

**Figure 5:**
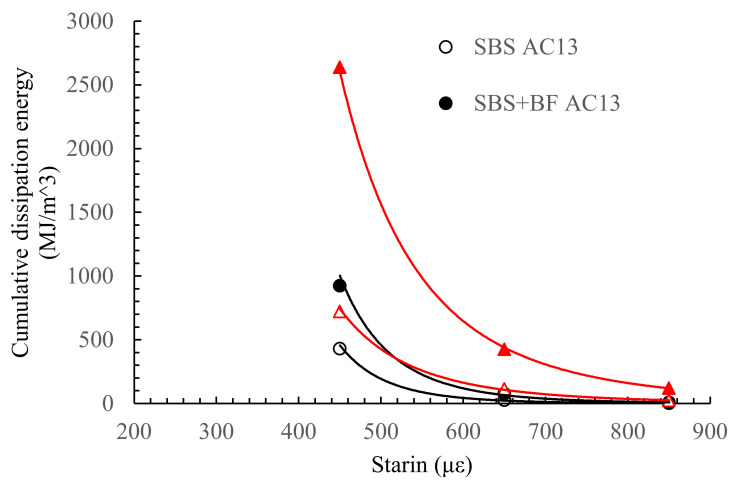
Cumulative dissipated energy results for the four-point bending fatigue test.

The fatigue life and cumulative dissipated energy results of the four-point bending fatigue test are shown in Figure 4 and Figure 5. The result is the average of five parallel measurements.

From Figure 4, the fatigue life of the four asphalt mixtures declines with the increasing levels of the control strain. A higher strain level causes more distress in asphalt mixtures, resulting in a lower fatigue life. Moreover, the addition of BF significantly increases the fatigue life of mixtures; for example, the fatigue life of AC-13 with and without BF is 264.7 × 10^3^ and 848.88 × 10^3^, where the latter is 3.2 times more than the former. Fibers can disperse the stress and reduce the stress concentration phenomenon in the asphalt mixture, resulting in a higher fatigue life. Another point that can be observed from Figure 4 is that the improvement of BF on fatigue life is about three times in AC and four times in SMA, indicating that the enhancement of BF is more significant in SMA mixtures. SMA is a skeleton-dense mixture, and AC is a suspension-dense mixture. Their different aggregate structures cause different synergistic effects with BF, leading to different enhancements in fatigue life.

Moreover, the fatigue life–strain relationship is fitted according to the classical equation, where *N* is the fatigue life, ε is the strain, and *A* and *m* are fitted parameters related to the material properties. The smaller the *m*, the more sensitive the fatigue life is to the strain level. The fitting results are shown in Figure 4. It can be seen that the fitted parameter *m* becomes smaller after BF is introduced to the mixtures. The value of *m* is 8.083 and 6.864 for AC-13 and SMA-13 without BF, and *m* is 7.946 and 6.430 after BF is introduced. This phenomenon indicates that BF reduces the sensitivity of the asphalt mixtures’ fatigue life to strain, which helps to improve the fatigue life of asphalt mixtures at high strain levels, allowing the asphalt mixtures to withstand heavier traffic loads.

In terms of energy dissipation, the overall trend in cumulative dissipation energy for the four asphalt mixtures is consistent. The cumulative dissipation energy decreases with the increasing levels of the control strain. The cumulative dissipation energy of asphalt mixtures also increases significantly with the addition of BF, increasing by 1.8 times in AC-13 and about 3.0 times in SMA-13. However, the trend of variation decreases. That is, the cumulative dissipation energy shows a decreasing trend with increasing strain control levels. However, it is clear that the dissipation energy increases somewhat with the addition of BF, indicating that the dissipation energy is not all caused by damage to the asphalt mixture itself, which indicates that the addition of BF reduces the energy consumed by damage to the mixture and improves the performance of the mixture by forming a three-dimensional mesh structure with uniform dispersion. The comparison of the trends of cumulative dissipation energy before and after the addition of BF shows that the latter is smaller than the former, implying that BF reduces the energy consumed due to damage to the mixture and thus prolongs the fatigue life of the mixture. Moreover, Figure 5 shows that the trend of the decreasing dissipation rate curves of the asphalt mixtures became slower with a longer stable decreasing phase after the addition of BF. The energy consumption per load cycle is higher with BF for the same micro-strain conditions, indicating that the asphalt mixtures with BF are more ductile and relatively challenging to deform.

Taken together, both AC-13 and SMA-13 show a significant improvement in fatigue life and cumulative dissipation energy after the introduction of BF. In particular, BF is more effective within SMA-13. The fatigue life of SMA-13 and AC-13 decreases with the increasing strain control levels, regardless of whether BF is added or not, which is in accordance with some other researchers’ findings [31,32].

### 4.2. Indirect Tensile Test Results

**Figure 6:**
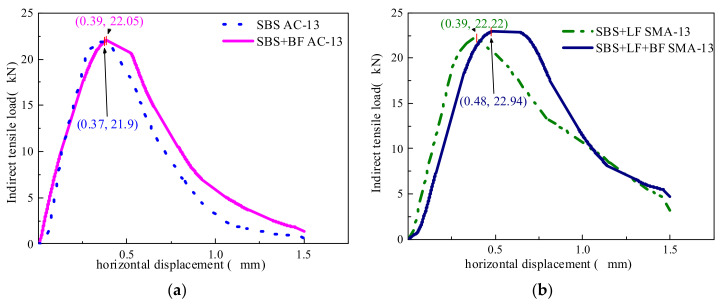
Horizontal displacement results of the indirect tensile test: (**a**) AC-13 and (**b**) SMA 13.

**Figure 7:**
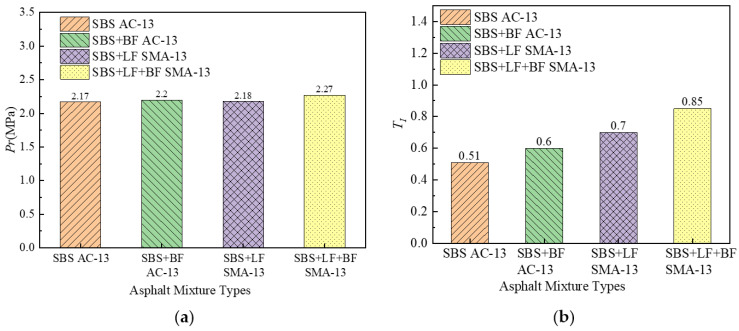
Indirect tensile test results: (**a**) tensile strength and (**b**) Resilience Index.

The results of the indirect tensile tests are shown in Figure 6 and Figure 7. As can be seen from Figure 6, the addition of BF to AC-13 and SMA-13 increases the maximum load value. The deformation curve of the asphalt mixture after the peak value tends to level off and decreases at a slower rate after the addition of BF, which indicates that the addition of BF improves the deformation resistance of the asphalt mixture. Fibers limit the development of cracks in the asphalt mixture, resulting in the asphalt mixture exhibiting a higher toughness. The reason for this fact is that the BF is probably distributed in the asphalt mixture to form a local spatial network structure, which transmits stress well and dissipates it, and effectively hinders the relative slip between the particles [33].

As can be seen from Figure 7, the increase in tensile strength of the asphalt mixture after the addition of fibers is not significant, and it does not appear to be ideal to judge the improvement of the BF on the cracking resistance of the asphalt mixture from the perspective of tensile strength. The tensile strength of mixtures with BF is only 2–4% higher than that without BF. However, the TI of the asphalt mixture increased substantially after the addition of BF. TI reflects the toughness of asphalt mixtures. The higher the TI, the better the toughness of the asphalt mixtures. A higher TI after the addition of BF indicates that BF can effectively improve the cracking resistance of the mixture. After the crack initiation, the BF in the asphalt mixture bears the more external load and reduces the load borne by the asphalt mixture matrix, retarding the development of the cracks. With the addition of BF, the TI of AC-13 and SMA-13 improved by 17.65% and 21.43%, respectively, indicating that the improvement of BF on asphalt mixture toughness is related to the mixture type, and the improvement of BF on SMA toughness is more remarkable than that on AC. This phenomenon is consistent with the fatigue test results. As previously discussed, basalt fibers can be stressed together with the asphalt mixture, which on the one hand allows the asphalt mixture to withstand greater external loads. On the other hand, the fibers retard the development of cracks in the asphalt mixture, allowing the asphalt mixture to exhibit higher cracking resistance.

### 4.3. Semicircular Bending Test Results

**Figure 8:**
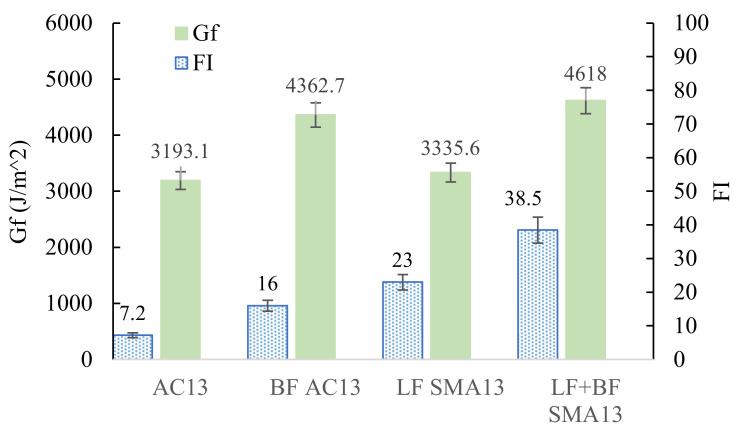
SCB test results.

The results of the SCB test are shown in Figure 8. The fracture energy, Gf, is significantly higher for the BF mixtures than the asphalt mixtures without BF. AC-13 with BF improved by approximately 36% compared to AC-13 without BF, and SMA-13 with BF increased by 38% compared to SMA-13 without BF. Gf is the fracture energy during the asphalt mixture cracking process. According to the fracture mechanics theory, the cracking of a material is a process of continuous energy consumption. The higher the fracture energy, the less likely the material is to crack. From the test results in Figure 8, it can be seen that basalt fibers significantly increase the Gf of the asphalt mixture, making the asphalt mixture less likely to crack. It is because basalt fibers form an inter-lap spatial network in the asphalt mixture, allowing the basalt fibers to share the external load with the asphalt mixture.

The variation pattern of FI is consistent with that of Gf. The FI with BF is significantly better than the FI without BF. The FI values of the AC-13 and SMA-13 increase by as much as two times and 67% with the introduction of BF. The cracking of the asphalt mixture is divided into two stages: the first stage is the sprouting of cracks, and the second stage is the expansion of cracks [34]. The FI characterizes the expansion ability of cracks. The experimental results showed that basalt fibers retarded the development of cracks, making microscopic cracks within the asphalt mixture less likely to develop into macroscopic cracks. As mentioned before, the BF acted as reinforcement during the cracking of asphalt mixtures. BF can form a network of inter-lap spaces to bear the load, which improves the stress concentration phenomenon inside the asphalt mixture and retards the crack development, resulting in improved cracking performance of asphalt mixtures.

### 4.4. Microscopic Morphology of Fiber Asphalt Mixture Sliced

**Figure 9:**
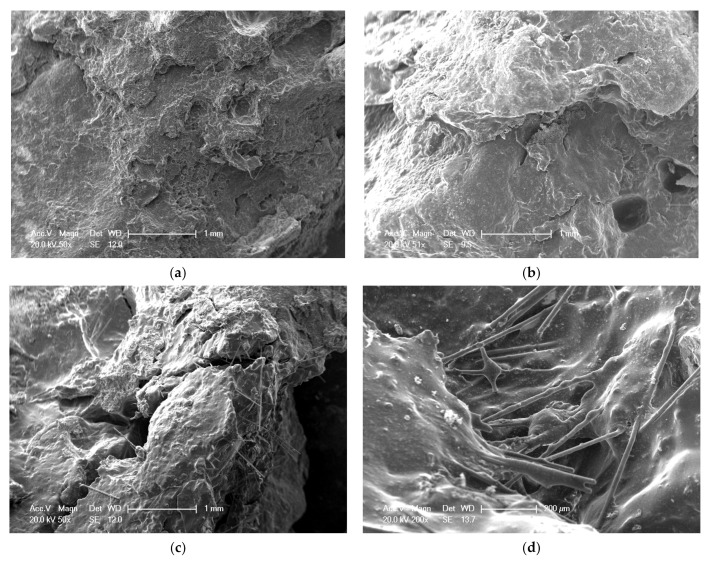
Microscopic images of asphalt mixtures: (**a**) SBS AC-13 (50×), (**b**) SBS+LF SMA-13 (50×), (**c**) SBS+BF AC-13 (50×), and (**d**) SBS+BF AC-13 (200×).

The damaged surface microstructure of the basalt fiber asphalt mixtures was observed with ESEM, and the results are shown in Figure 9. Figure 9a,b are the microscopic images of AC-13 and SMA-13 without BF at a magnification of 50×. Figure 9c,d are at the microscopic images of BF AC-13 at a magnification of 50× and 200×, respectively.

As shown in Figure 9a, the asphalt mixture without BF has a relatively flat section with a certain degree of peeling. Moreover, by comparing Figure 9a,b, it can be seen that BF has no significant effect on the microscopic image of the asphalt mixture. In Figure 9b, the microscopic images of SMA-13 with LF, LF is barely visible in the mixture, indicating that LF only plays a role in absorbing the extra free asphalt in the asphalt mixture without significantly improving the mechanical properties of the asphalt mixture [35]. In contrast, BF can be easily observed in Figure 9c, and a clearer supporting microscopic image is shown in Figure 9d. According to the composite theory, adding fiber is an effective method to improve the strength and toughness of materials [36]. The microscopic pictures of BF asphalt mixtures show that BFs are coated with asphalt, indicating that the adhesion between BFs and asphalt is good, which is conducive to the cooperative work of the fiber and asphalt mixture. Moreover, the section of the asphalt mixture with BF shows that numerous BFs are randomly dispersed in the asphalt mixture in three dimensions and lapped together to form a spatial network. BF’s strength and modulus are relatively higher than asphalt mixtures, so BFs bear more loads than asphalt mixtures. This fiber network limits the movement of asphalt and aggregate and increases the asphalt mixture strength, making the asphalt mixtures less prone to cracking. At the same time, once the asphalt mixture sprouts cracks, this fiber network can improve the toughness of asphalt mixtures by delaying the development of cracks. Thus, BFs effectively improve the crack resistance of the asphalt mixture. Furthermore, the fatigue test results imply that the effect of the BF network within asphalt mixtures is strain dependent, and BF improves the fatigue life more at a higher strain level. As discussed previously, the modulus of BF is relatively higher than that of asphalt mixtures; thus, when a fibrous asphalt mix is stressed, the asphalt mix matrix deforms first and transfers the force to the fiber network. The higher the strain on the asphalt matrix, the greater the force shared by the fiber network and the more pronounced the strengthening effect of the fiber network.

**Figure 10:**
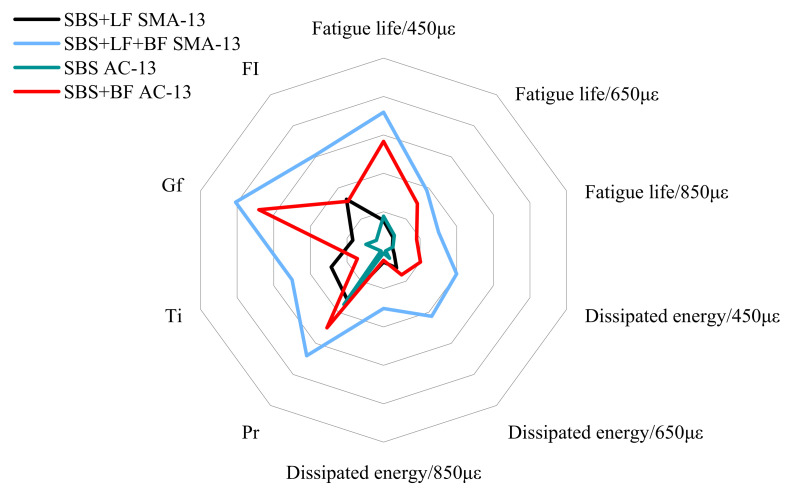
Crack resistance of different asphalt mixtures.

Figure 10 provides a comprehensive demonstration of the cracking resistance of BF in different asphalt mixtures. As shown in Figure 10, there is a clear difference in the BF improvement on the crack resistance of different types of asphalt mixtures. From all aspects of the analysis, SMA-13 has the best crack resistance, partly due to the reinforcing effect of basalt fibers and partly because the higher amount of asphalt in SMA facilitates the crack resistance of the asphalt mixture. From Figure 10, it can be observed that BFs provide a greater enhancement to the performance of SMA-13. Therefore, besides well-known factors such as BF content, the type of asphalt mixture and the fit of the BF also exert a vital influence, which should be paid attention to by engineers when selecting the type of asphalt mixture.

## 5. Conclusions

In this study, the influence of BF on the crack resistance of asphalt mixtures was evaluated. Based on the previous analysis and discussion, the following conclusions can be drawn:

(1) The addition of BF significantly increased the fatigue life and the accumulated dissipation energy of the mixture, by up to 3~4 times in AC-13 and by about 4~5 times in SMA-13, resulting in asphalt mixtures being less prone to fatigue cracking.
(2) After adding BF, the indirect tensile strength of the asphalt mixture slightly increased. The TI substantially increased. BF significantly increased the toughness and improved the anti-cracking properties of the mixture.
(3) BF improved both the FI and Gf in the SCB test, indicating that BF can delay the development of cracks.
(4) BFs were randomly dispersed in the asphalt mixture and lapped together to form a spatial network. This fiber network made asphalt mixtures less prone to cracking and delayed the development of cracks.
(5) The reinforcing effect of BF is related to aggregate gradation and the strain level. The reinforcing effect of BF was more pronounced for SMA13. Moreover, the higher the strain in the asphalt mix matrix, the greater the reinforcing effect of BF.

In the future work, more evaluation of the interaction between BF and the asphalt mixture is needed to further clarify the reinforcement mechanism of BF.

## Figures and Tables

**Table 1:** Results of SBS asphalt properties.

Index	Specification Requirements	Value	Test Method
Penetration at 25 °C/0.1 mm	60~80	71	ASTM D 5
Softening point/°C	≮55	64	ASTM D 2398
Ductility at 5 °C/cm	≮30	48	ASTM D 113
Viscosity at 135 °C/Pa.s	≯3	1.8	ASTM D 4402
Elastic recovery at 25 °C/%	≮65	76	ASTM D 6084

**Table 2:** Properties of modifiers.

	Types	BF	LF
Characteristics			
Color		Golden brown	Gray
Form		Smooth	Loose flocculent
Single fiber diameter/μm		13~16	≈13
Length/mm		6	0.8
Density/(g·cm^−3^)		2.715	1.295
Breaking strength/Mpa		≥2000	<300
Melting point/°C		1600	230

**Table 3:** Results of aggregate properties.

Aggregate Size (mm)	10–15 mm		5–10 mm		3–5 mm		0–3 mm	
	Limestone	Basalt	Limestone	Basalt	Limestone	Basalt	Limestone	Basalt
Bulk relative density	2.753	2.831	2.746	2.807	2.721	2.886	2.635	2.895
Apparent relative gravity	2.776	2.931	2.778	2.936	2.768	2.927	2.695	2.967
Water absorption (%)	0.30	0.12	0.42	0.16	0.62	0.48	0.84	0.82

**Table 4:** Volume parameters of AC-13 and SMA-13.

Items	Optimal Asphalt Content (OAC) (%)		Voids Volume (VV) (%)		Voids in the Mineral Aggregate (VMA) (%)		Voids Filled with Asphalt (VFA) (%)	
	AC-13	SMA-13	AC-13	SMA-13	AC-13	SMA-13	AC-13	SMA-13
Without BF	>4.7	>5.8	>4.1	>3.8	>14.2	>17.2	>71.1	>77.9
With BF	4.9	5.5	4.1	3.9	14.3	16.7	71.3	76.6
Specification	-	-	3~6	3–4	≮14.0	≮16.5	65~75	75–85

## Data Availability

The data presented in this study are available from the corresponding author upon request.
